# Vascular density and phenotype around ductal carcinoma *in situ* (DCIS) of the breast

**DOI:** 10.1038/sj.bjc.6600053

**Published:** 2002-03-18

**Authors:** N B Teo, B S Shoker, C Jarvis, L Martin, J P Sloane, C Holcombe

**Affiliations:** Pathology Department, University of Liverpool, Duncan Building, Daulby Street, Liverpool L69 3GA, UK; Breast Unit, Linda McCartney Centre, Royal Liverpool University Hospital, Prescot Street, Liverpool L7 8XP, UK

**Keywords:** breast, ductal carcinoma *in situ*, angiogenesis, endothelial phenotype, invasive carcinoma

## Abstract

Up to 50% of recurrences of ductal carcinoma *in situ* of the breast are associated with invasive carcinoma but no pathological or molecular features have yet been found to predict for the development of invasive disease. For a tumour to invade, it requires the formation of new blood vessels. Previous studies have described a vascular rim around ducts involved by ductal carcinoma *in situ*, raising the possibility that the characteristics of periductal vascularisation may be important in determining transformation from *in situ* to invasive disease. Periductal vascular density and phenotype were determined using morphometry and a panel of anti-endothelial antibodies (von Willebrand factor, CD31, CD141 and CD34) and related to the presence of invasive carcinoma and other histological features. Compared to normal lobules, pure ductal carcinoma *in situ* exhibited a greater density of CD34^+^ and CD31^+^ vessels but a decrease in those that were immunopositive for vWF, indicating a difference in phenotype and in density. Ductal carcinoma *in situ* associated with invasive carcinoma showed a profile of vascular immunostaining similar to that of pure ductal carcinoma *in situ* but there were significantly greater numbers of CD34^+^ and CD141^+^ vessels and fewer staining for vWF. There was a significant negative correlation between vascular density and both the cross-sectional areas of the ducts involved and the extent of the necrosis of the tumour they contained. A correlation between vascular density and nuclear grade was also noted, being highest in the intermediate grade. The greater density of CD34^+^ and CD141^+^ vessels around ductal carcinoma *in situ* associated with invasive carcinoma could reflect a greater predisposition to invade but a direct effect of co-existent invasive carcinoma cannot entirely be ruled out in the present study. The relationship between vascular density, grade, duct size and nuclear grade suggests that periductal angiogenesis increases with tumour growth rate but is unable to keep pace with the most rapidly growing lesions.

*British Journal of Cancer* (2002) **86**, 905–911. DOI: 10.1038/sj/bjc/6600053 www.bjcancer.com

© 2002 Cancer Research UK

## 

With the advent of screening mammography, the frequency with which ductal carcinoma *in situ* (DCIS) is detected has increased from approximately 1 to 20% of all breast cancers and is as high as 30% in some centres (Nemoto *et al*, 1980; Fryberg and Bland, 1994). Histological grade, lesion size and excision margin status are strongly related to the probability of developing local recurrence (Recht *et al*, 1985; Silverstein *et al*, 1995; Badve *et al*, 1998) and important in determining management. Recurrent *in situ* carcinoma is itself innocuous but up to 50% of recurrences are associated with invasive carcinoma (Recht *et al*, 1985; Silverstein *et al*, 1995; Badve *et al*, 1998). To date, no pathological or molecular features have been found to predict for the development of invasive disease.

For a tumour to grow beyond 1–2 mm^3^, it requires the formation of new blood vessels to supply oxygen and nutrients as well as to excrete catabolites. There have been many studies of the process of angiogenesis in breast carcinoma but most have focused on invasive disease. In DCIS and other precursor lesions (Guidi *et al*, 1994; Engels *et al*, 1997a,b; Lee *et al*, 1997; Lee *et al*, 1999; Sales *et al*, 1999; Valtola *et al*, 1999), two patterns of vascularity have been described: diffuse stromal vascularity and a vascular rim around the involved ducts. Pre-malignant lesions of the breast can induce angiogenesis in animal experimental systems and in the human breast (Gimbrone and Gullino, 1976; Brem *et al*, 1977; Heffelfinger *et al*, 1996; Lichtenbeld *et al*, 1998), it thus seems possible that the pattern or extent of vascularisation around DCIS may be an important factor in determining the transformation from *in situ* to invasive carcinoma. It is likely that the periductal vessels are most important in this respect as incipient invasion is most likely to be associated with changes in vessels in the immediate vicinity of the tumour cells.

The main aim of this study was to test the hypothesis that the development of invasive carcinoma in DCIS is associated with changes in periductal vessels. The phenotype and number of microvessels were compared in pure DCIS with those in DCIS associated with invasive carcinoma. As studies of breast cancer angiogenesis in the past have been associated with inconsistent findings, we have used precise morphometric methodology to compare vascularity in DCIS with normal breast and have employed a panel of anti-endothelial antibodies to take account of phenotypic as well as numerical changes. At the same time we have taken the opportunity to determine if vascular density and phenotype have any relationship to histological features, particularly nuclear grade, necrosis and duct size.

## MATERIALS AND METHODS

### Patients and tumours

Formalin-fixed paraffin-embedded breast samples (*n*=40) of patients with ductal carcinoma *in situ*, with (*n*=20) and without (*n*=20) invasive carcinoma, were collected from the archives of the Pathology Department of the Royal Liverpool University Hospital. The original haematoxylin and eosin (H&E) stained sections of the primary tumours were reviewed by two pathologists (BS Shoker and JP Sloane) for classification by architecture and nuclear grade following the guidelines of the European Commission and UK National Breast Screening Programme (National co-ordinating group for breast screening pathology 1997, The consensus conference committee 1997). A representative block for each patient was selected for subsequent immunostaining.

### Immunohistochemistry

Sections were stained for endothelial cells using monoclonal anti-CD31 (JC/70A, DAKO, Denmark), anti-CD34 (Qbend/10, DAKO, Denmark), anti-CD141 (Anti-Thrombomodulin, DAKO, USA) and polyclonal anti-human von Willebrand factor (vWF) (DAKO, Denmark).

Sections were dewaxed through two changes of xylene and industrial methylated spirits (IMS). Endogenous peroxidase activity was blocked with a mixture of H_2_O_2_/methanol (12 ml H_2_O_2_ in 400 ml methanol) for 12 min. Antigen retrieval was performed for the endothelial markers by treating the sections with 0.2 g of trypsin and 0.4 g of calcium chloride in 440 ml Tris Buffered Saline (TBS (50 mM Tris-HCl, 150 mM NaCl, pH 7.4)) at 37°C for 20 min. For Ki67 antigen retrieval was performed by immersing the sections in 2000 ml of Ethylenediamine-Tetraacetic Acid (Sigma Chemical, St Louis USA) and then boiling in a pressure cooker for 8 min. Prior to staining with the polyclonal antiserum, sections were treated with a mixture of 5% bovine serum albumen (BSA)/TBS (1 g Bovine Serum Albumen in 20 ml TBS) for 10 min.

The antibodies were diluted 1 : 5 for anti-CD31, 1 : 10 for anti-CD141, 1 : 20 for anti-CD34 and 1 : 1000 for anti-human vWF and Ki67 in 5% BSA/TBS. The sections were incubated with primary antibodies at room temperature for 40 min. Secondary antibodies were incubated for 40 min using EnVision Labelled Polymer (mouse or rabbit as appropriate). Sections were washed with TBS between incubation steps. 33′diaminobenzidine, DAB was used as a chromogen. The last two steps were carried out using a commercial kit (DAKO EnVision TM^+^ System, Peroxidase (DAB), USA).

The cell nuclei were counter-stained with Haematoxylin in solution. The sections were dehydrated through four changes of IMS and three changes of xylene before being mounted in resinous mountant (DPX, BDH Laboratory supplies, UK). Omission of the primary antibody was used as a negative control and the microvessels of the normal adjacent breast tissue served as internal positive controls.

### Assessment of tumour vascularity

NB determined vascular density without knowing how the architecture or nuclear grade had been classified. No scanning of stained microvessels to identify ‘hot-spots’ at low magnification was undertaken. The assessment of completely transected involved ducts started from the upper right of all stained sections, moving downwards and to the left. All or the first 50 foci (duct cross sections) encountered were assessed on each section, thus eliminating selection bias.

In the assessment of DCIS accompanied by invasive carcinoma, only those foci of DCIS that were at least 2 mm away from the invasive component were evaluated to minimise detecting direct local effects of the invasive disease.

### Evaluation of DCIS area

**Figure 1:**
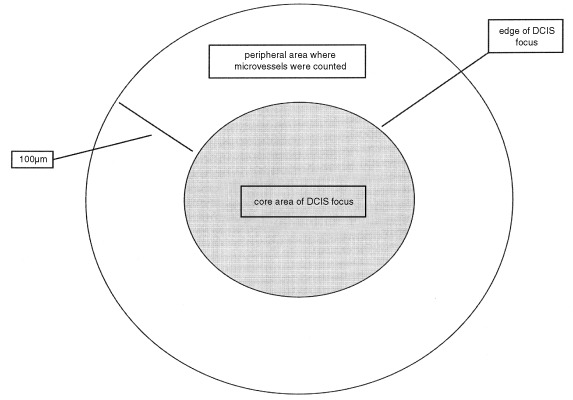
Simplified diagram of an individual focus of ductal carcinoma *in situ* (DCIS) and peripheral area where the periductal microvessels were counted.

The area of each individual focus of DCIS was measured at ×200 magnification using an image analysis system (Zeis Axiohome with software version 3.0, Germany). In addition, the area with a circumference 100 μm from the edge of an individual focus of DCIS was measured. The area required for MVD assessment was thus calculated by subtracting the former from the latter (Figure 1

).

### Counting microvessels

**Figure 2:**
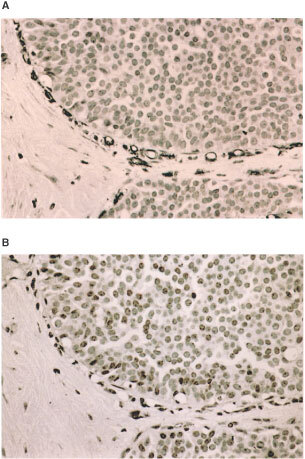
(**A**) Ductal carcinoma *in situ* (DCIS) stained immunohistochemically using the CD34 antibody revealing a rim of positive vessels, (**B**) same area stained for vWF. There is a striking difference in the number of positive cells with the two antibodies in this case.

The microvessels within 100 μm around each DCIS focus were counted at high magnification (×400). Eligible microvessels included any immunostained endothelial cell or cluster of cells around a visible lumen clearly separated from adjacent microvessels, tumour cells and other connective tissue components (Figure 2

). The presence of red blood cells was not required. It was not possible to distinguish blood and lymphatic vessels. Where vessels were in clusters, each was counted as separate if it met the above criteria.

### Controls

Normal breast lobules were used as internal controls and up to five normal lobules were assessed for each case of DCIS. The normal lobules were assessed only if they were situated more than 2 mm from the nearest tumour.

### Evaluation of degree of necrosis

As part of the histological assessment, the degree of necrosis was semi-quantitatively assessed in sections of high grade DCIS from patients with and without invasive disease. Each individual focus of DCIS was given a score of 1–3. A score of 1 was given when no necrosis was present in an individual focus of DCIS, a score of 2 when between 1 and 50% necrosis was present and a score of 3 when there was more than 50% necrosis.

The data for each duct space were analysed using the Kruskal-Wallis method and Mann-Whitney *U*-test and by the 2-tailed Spearman's or Pearson's Correlation coefficient using SPSS software (Version 10.0 for Window NT).

## RESULTS

The two groups of DCIS were matched for nuclear grade, each contained 7 low, 6 intermediate and 7 high nuclear grade cases.

### Microvessel density in DCIS without invasive carcinoma

#### Normal lobules

The highest values of MVD were obtained using vWF followed by CD34, CD141 and CD31. The mean MVD was significantly different between antibodies (Mann-Whitney, highest *P*=0.01) apart from between CD141 and CD34 (Mann-Whitney, *P*=0.28). (Table 1

**Table 1:**
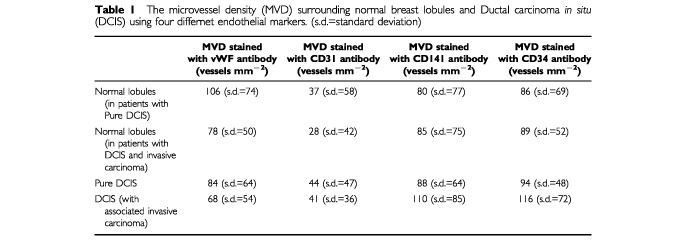
The microvessel density (MVD) surrounding normal breast lobules and Ductal carcinoma *in situ* (DCIS) using four differnet endothelial markers. (s.d.=standard deviation)

; Figure 3

**Figure 3:**
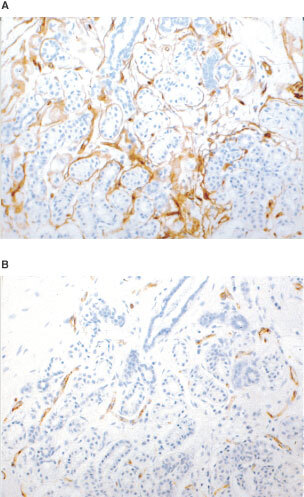
(**A**) Non-cancerous lobules stained immunohistochemically using the CD34 antibody revealing a rim of positive vessels, (**B**) same area stained for vWF. There is also a striking difference in the number of positive cells with the two antibodies in this case.

).

#### DCIS

For DCIS the highest MVD was obtained using the antibody to CD34, followed by CD141, vWF and CD31. In comparison to normal lobules there were increases in CD34, CD141 and CD31 positive vessels but a decrease in those immunopositive for vWF. Changes in MVD detected by the CD34, CD31 and vWF antibodies were statistically significant (Mann-Whitney, highest *P*=0.015) (Table 1). MVD around DCIS using CD34 was less than that around normal lobules using anti-vWF. However, the difference was not statistically significant (Mann-Whitney, *P*=0.35).

### Microvessel density in DCIS with invasive carcinoma

#### Normal lobules

In normal lobules surrounding cases of pure DCIS, MVD was highest when stained with vWF, whereas the normal lobules surrounding areas of invasive carcinoma and DCIS had the highest MVD with CD34. However, the only significant differences between the antibodies were that CD31 gave lower values than the other three (Mann-Whitney, highest *P*<0.001) (Table 1). The MVD in normal lobules with DCIS and invasive cancer obtained with vWF was significantly lower than in normal lobules from cases of pure DCIS (Mann-Whitney, *P*=0.014) whereas those obtained with CD31, CD34 and CD141 showed no significant difference (Mann-Whitney, lowest *P*=0.2).

#### DCIS

In DCIS, the highest MVD was obtained using the CD34 antibody. There were significant differences among all four antibodies (Mann-Whitney, highest *P*=0.034). (Table 1). In DCIS with invasive carcinoma, in common with cases of pure DCIS, there was an increase above normal in CD31, CD34 and CD141 but a decrease in vWF immunopositive vessels. All these changes were statistically significant (Mann-Whitney, highest *P*=0.03). When DCIS with invasive cancer compared with *pure* DCIS, we showed an increase in MVD as determined by CD34 and 141 and decreases as determined by CD31 and vWF. These findings were all statistically significant (Mann-Whitney, highest *P*=0.001) except for CD31 (Mann-Whitney, *P*=0.3).

### Relationship between MVD and nuclear grade

**Figure 4:**
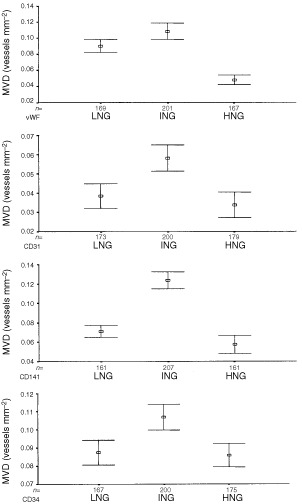
Relationship between microvessel density (MVD) and nuclear grade in ductal carcinoma *in situ* (DCIS) without invasive carcinoma using four types of endothelial markers. The boxes represent the mean values and the whiskers represent 95% confidence interval of the mean. (LNG=low nuclear grade, ING=intermediate nuclear grade, HNG=high nuclear grade).

MVD increased from low to intermediate nuclear grade but decreased in high grade DCIS usually to a level below that of low grade. The difference between low and intermediate grade DCIS was statistically significant for pure and invasive cases using all antibodies (Mann-Whitney, highest *P*=0.007) except for pure DCIS when MVD was determined using vWF (Mann-Whitney, *P*=0.07). The difference between intermediate and high grade was significant for both pure and invasive cases of DCIS with all antibodies (Mann-Whitney, highest *P*<0.001) except for invasive cases when MVD was determined by CD31 (Mann-Whitney, *P*=0.4). The difference between low and high grade, however, was significant only for pure DCIS using CD141, CD31 and vWF antibodies and for invasive cases using CD31 (Mann-Whitney, highest *P*=0.047) (Figure 4

).

### Relationship between MVD and core area

**Figure 5:**
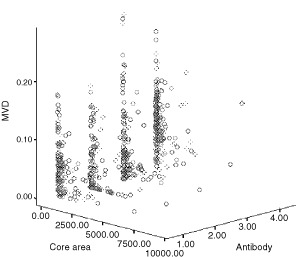
Relationship between microvessel density (MVD) and core area in ductal carcinoma *in situ* (DCIS) without invasive carcinoma using four types of endothelial markers. (1=vWF, 2=CD31, 3=CD141, 4=CD34).

For all cases of DCIS the MVD fell with increasing size of the individual focus of DCIS. This negative correlation was statistically significant as determined with all four antibodies (Spearmans, highest *P*<0.001) except when CD31 was used on cases of DCIS associated with invasion (Spearmans, *P*=0.3) (Figure 5

).

### Relationship between MVD, core area and necrosis

**Figure 6:**
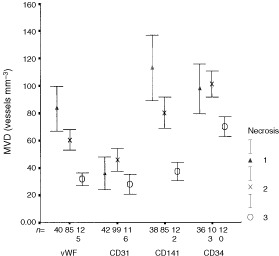
Relationship between microvessel density (MVD) and degree of necrosis in ductal carcinoma *in situ* (DCIS) with and without invasive carcinoma. The boxes represent the mean values and the whiskers represent 95% confidence interval of the mean. (Degree of necrosis: (1) no necrosis, (2) 1–50% of DCIS focus has necrosis, (3) >50% of DCIS focus has necrosis).

As these analyses were restricted to high nuclear grade DCIS, cases with and without invasive carcinoma were pooled. A significant negative correlation was observed between necrosis scores and MVD using all four antibodies (Spearmans, highest *P*=0.002) (Figure 6

).

**Figure 7:**
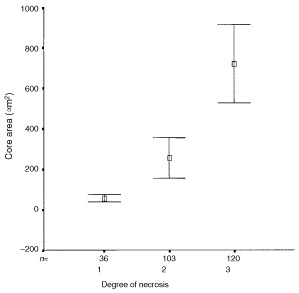
Relationship between degree of necrosis and core area in ductal carcinoma *in situ* (DCIS) with and without invasive carcinoma. The boxes represent the mean values and the whiskers represent 95% confidence interval of the mean. (Degree of necrosis: (1) no necrosis, (2) 1–50% of DCIS focus has necrosis, (3) >50% of DCIS focus has necrosis).

A significant positive correlation was also observed between necrosis scoring and core area of the foci for both pure DCIS and DCIS associated with invasion (Spearmans, highest *P*<0.001) (Figure 7

).

## DISCUSSION

Angiogenesis has been shown to have prognostic significance in breast cancer (Weidner *et al*, 1991; Bosari *et al*, 1992; Horak *et al*, 1992; Weidner *et al*, 1992) and several studies have shown that pre-malignant lesions of the breast can induce angiogenesis in animal experimental systems and in human breast (Gimbrone and Gullino, 1976; Heffelfinger *et al*, 1996; Brem *et al*, 1977; Lichtenbeld *et al*, 1998). In 1976, Gimbrone *et al* (1976) showed that 30% of implants from pre-malignant murine hyperplastic alveolar nodules showed angiogenesis on the irises of New Zealand White rabbits. Furthermore, tissues of higher predicted tumour incidence induced greater neo-vascular responses than did those with a lower probability of malignant transformation. These findings have been confirmed in a number of similar studies (Guinebretiere *et al*, 1994; Brem *et al*, 1977; Lichtenbeld *et al*, 1998). We therefore felt that it was reasonable to hypothesize that changes in periductal vascularity may precede the development of invasive carcinoma in DCIS.

Two patterns of angiogenesis have been described in DCIS: a diffuse stromal vascularity (pattern I) and a vascular rim around the involved ducts (pattern II) (Guidi *et al*, 1994). Subsequently, it was proposed that pattern II resulted from angiogenic factors secreted by tumour cells and pattern I from recruitment of accessory cells, which release angiogenic factors (Engels *et al*, 1997a). In our study, we restricted our investigations to vascular pattern II as we hypothesized that incipient invasion is likely to be associated with vascular changes in the immediate vicinity of the tumour cells.

In the present study the different antibodies used gave different values for MVD both in normal lobules and DCIS indicating that they identified different sub-populations of small vessels. In pure DCIS, there was a significant increase above normal in CD34^+^ and CD31^+^ vessels but a reduction in those staining for vWF. These findings are in keeping with a change in phenotype as well as vascular density.

In DCIS associated with invasive carcinoma, there were significantly greater numbers of CD34^+^ and CD141^+^ vessels and fewer staining for vWF compared with pure DCIS. It is not clear whether this higher periductal MVD reflects a greater predisposition to invade or whether the effect is due to the proximity of invasive carcinoma. We attempted to minimize the latter possibility by evaluating DCIS at least 2 mm from the nearest invasive carcinoma but this would not exclude any effect of factors released by the tumour into the blood or lymphatic system. It is of interest in this context that the normal lobules from cases of invasive carcinoma exhibited significantly lower numbers of vWF^+^ vessels than those from cases of pure DCIS. This finding is in apparent conflict with the work of Heffelfinger *et al* (1996) who found the vascularity of histopathologically normal epithelium to be greater in breast containing invasive carcinoma using an antibody to vWF. The breasts without invasive disease in this study, however, included those with benign proliferative change as well as DCIS.

The different antibodies used gave different values for MVD in normal breast and showed dissimilar changes in DCIS. For patients with and without invasive disease, vWF^+^ vessels were at lower density in DCIS than in the normal breast. This is in keeping with observations on invasive tumours (colorectal carcinoma) where vWF immunostaining has been found to be absent from some of the capillaries in the tumour (Vermeulen *et al*, 1995). Anti-vWF stains large vessels more strongly than small ones (Vermeulen *et al*, 1996) and consequently our findings could reflect the immaturity of newly formed tumour-associated vessels. The density of CD34^+^ vWF^-^ or CD141^+^/vWF^-^ vessels could thus reflect the rate tumour angiogenesis and the consequences it has for the biological behaviour of DCIS.

CD31 gave consistently lower values for MVD than the other antibodies. The molecule has a role in platelet adhesion in inflammation and wound healing (Parums *et al*, 1990) and is expressed on large and small vessels in either normal or tumour tissue (Horak *et al*, 1992; Toi *et al*, 1993; Vermeulen *et al*, 1996). In contrast to vWF it appears not to be expressed on lymphatic vessels (Burgdorf *et al*, 1981) and this could at least partially explain why determinations of MVD with the CD31 antibody were consistently lower in both normal lobules and DCIS. The possible contribution of lymphatics to neo-vascularisation in tumours has been largely ignored.

Values for MVD as determined using CD34 and 141 antibodies were roughly comparable both in normal lobules and DCIS. Both showed a significant increase in DCIS, with greater values in cases associated with invasive carcinoma. Our findings confirm those of Martin *et al* (1997), that CD34 antibodies give consistently higher vessel counts in breast carcinomas than antibodies against vWF or CD31.

We found an unexpected relationship between MVD and nuclear grade of DCIS. In those with and without invasive disease, the MVD was significantly higher in intermediate than either low or high-grades. There was also a significant negative correlation between vascular density and both the cross-sectional areas of the ducts involved and the extent of the necrosis of the tumour they contained. The last finding is in apparent conflict with the study of Guidi *et al* (1994), who found that comedo-type lesions were more likely to be associated with higher microvessel density. In their study, however, vessels up to 500 μm from the involved structures were included and only those from the five most vascular fields were recorded. Vessels forming cuffs around ducts were not studied quantitatively as they were found only in a minority of cases. These authors, however, identified vessels using an antibody to vWF only, which in our study stained significantly fewer vessels than the CD34 and CD141 antibodies. A likely explanation for our findings is that angiogenesis increases with growth rate but is unable to keep pace with the most rapidly growing lesions. The rapidly proliferating lesions tended to contain larger foci of DCIS and often showed comedo necrosis.

In conclusion, the greater density of CD34^+^ and CD141^+^ vessels around DCIS associated with invasive carcinoma could reflect a greater predisposition to invade but a direct effect of co-existent invasive carcinoma cannot entirely be ruled out in the present study. The relationship between vascular density, grade, duct size and nuclear grade suggests that periductal angiogenesis increases with tumour growth rate but is unable to keep pace with the most rapidly growing lesions.
